# Effectiveness of digital health education interventions for chronic disease self-management in community-dwelling older adults: an umbrella review and evidence classification

**DOI:** 10.3389/fpubh.2026.1948010

**Published:** 2026-10-12

**Authors:** Esra Sezer, Gözde Gür

**Affiliations:** Department of Nursing, Faculty of Health Sciences, Acibadem Mehmet Ali Aydinlar University, Istanbul, Türkiye

**Keywords:** chronic disease, community-dwelling older adults, digital health education, self-management, umbrella review

## Abstract

**Background:**

Digital health education interventions are widely promoted to support chronic disease self-management. However, existing umbrella reviews pool evidence across all ages and have not assessed community-dwelling older adults, who face barriers to digital access. This umbrella review aimed to determine whether current evidence supports the effectiveness of digital health education interventions for chronic disease self-management in community-dwelling adults aged 60 years or older.

**Methods:**

We conducted an umbrella review of systematic reviews evaluating digital health education interventions for chronic disease self-management in community-dwelling adults aged 60 years or older. Six databases were searched for systematic reviews published between 2015 and 2026. Meta-analytic outcomes were re-estimated using random-effects models, evidence was graded according to the Ioannidis criteria, methodological quality was assessed with AMSTAR 2, and primary-study overlap was quantified using the corrected covered area (CCA).

**Results:**

Of the 1,272 records identified, 11 met the eligibility criteria, and 10 contributed 44 meta-analytic outcomes derived from 199 primary randomized controlled trials (RCTs) across five chronic disease clusters. The evidence base showed minimal overlap among primary studies (overall CCA was 0.76%). None of the outcomes met the criteria for convincing (Class I), highly suggestive (Class II), or suggestive (Class III) evidence. Fifteen outcomes (34.1%) were classified as weak (Class IV), whereas 29 (65.9%) were non-significant. According to AMSTAR 2, only three of the 10 systematic reviews were rated as high confidence, whereas two were rated as low and five as critically low confidence; the 95% prediction interval crossed the null for all 44 outcomes.

**Conclusion:**

When the evidence is limited to community-dwelling older adults, no digital health education intervention for chronic disease self-management is supported by high-credibility evidence at the umbrella review level. This finding most likely reflects the immaturity of the current evidence base—characterized by a limited number of trials, short follow-up periods, and substantial heterogeneity—rather than a lack of intervention effectiveness. Well-powered RCTs with longer follow-up and higher-quality systematic reviews specifically focused on older adults are needed to strengthen the evidence base.

## Introduction

The global population is aging at an unprecedented rate, with the number of older adults increasing both in absolute terms and as a proportion of the total population. According to the United Nations World Population Prospects, the proportion of people aged 65 years or older is projected to increase from 10% in 2022 to 16% in 2050 (1). This demographic transition is driven by declining mortality, increasing life expectancy, and sustained reduction in fertility rates. By 2050, more than 80% of adults aged 60 years or older are expected to live in low- and middle-income countries (1). As health systems adapt to this demographic shift under growing service demand and workforce constraints, scalable strategies are needed to extend chronic-disease management and self-care support to older populations—an aim for which digital health has been increasingly promoted (2).

Population aging coincides with the increasing disability burden of noncommunicable diseases (NCDs), making the preservation of healthy life-years a priority for health systems (1). In 2021, NCDs caused at least 43 million deaths worldwide, accounting for 75% of all deaths. Cardiovascular diseases were the leading cause of NCD-related deaths, followed by cancers, chronic respiratory diseases, and diabetes. Nearly three-quarters of NCD-related deaths occurred in low- and middle-income countries. NCDs are influenced by behavioral factors (tobacco use, unhealthy diet, harmful alcohol use, and physical inactivity), metabolic factors (high blood pressure, overweight or obesity, elevated blood glucose, and dyslipidemia), and environmental factors (air pollution) (3). Reducing modifiable risk factors and supporting effective disease management are key strategies for reducing the burden of NCD. As a result, older adults must play an active role in managing their own health, making effective self-management interventions essential for both disease prevention and rehabilitation.

Lorig and Holman proposed that self-management education equips individuals with the problem-solving, decision-making, resource utilization, patient–health care professional partnership, and action-planning skills needed to manage the medical, role, and emotional aspects of chronic disease. These skills facilitates behavior change by enhancing self-efficacy (4). Self-efficacy is considered the principal mechanism underlying the educational interventions examined in this study.

The World Health Organization Global Strategy on Digital Health 2020–2025 recognizes that digital technologies, including virtual care, remote monitoring, and wearable devices, can improve health outcomes, including chronic disease self-management, and recommends expanding access to evidence-based self-management tools (5). In this study, digital health education interventions include mobile health applications, telehealth and telemonitoring, wearable devices, web-based programs, text messaging, telecare, internet-based coaching, chatbot- or other artificial intelligence-based tools, and digital self-monitoring platforms that incorporate an educational component.

The coronavirus disease 2019 (COVID-19) pandemic markedly accelerated the adoption of telehealth applications, driving lasting structural changes in health care systems (6). Although older adults are among the populations most likely to benefit from these services, interventions tailored to their specific needs remain inadequately developed (7). Wildenbos et al. reported that age-related barriers—including cognitive and physical limitations, sensory impairments, and reduced motivation—may negatively affect the usability of digital health technologies (8). Beyond limiting usability, these barriers may also act as effect modifiers: by constraining sustained engagement, comprehension of educational content, and the behavior change on which self-management depends, they could attenuate the treatment effect of digital health education interventions in older adults relative to younger populations. This possibility provides the rationale for evaluating effectiveness specifically in community-dwelling older adults, in whom pooled effect sizes may be smaller or null.

Numerous systematic reviews have evaluated digital interventions in chronic disease management, including computer-based self-management interventions for diabetes (9), structured telephone support and noninvasive telemonitoring in heart failure (10), and digital interventions for hypertension (11). However, these reviews have generally focused on a single disease or intervention modality, resulting in fragmented and sometimes inconsistent findings. An umbrella review provides a high-level approach to synthesizing evidence from published systematic reviews addressing a common research question; however, the strength of its conclusions depends on the methodological quality and certainty of the underlying systematic reviews and primary studies (12). A recent umbrella review of mobile phone–based interventions for chronic diseases identified convincing (Class I) evidence for only a single outcome—reduction in glycated hemoglobin in type 2 diabetes—across 34 meta-analyses, and did not establish high-level evidence for most outcomes (13). Older adults who live in the community—that is, in their own homes rather than in institutional, long-term care, or hospital settings, irrespective of their level of functional independence—constitute the population of primary interest, because they must self-manage chronic disease with limited on-site clinical support and are the group in whom digital health is most often proposed as a scalable solution. To our knowledge, no umbrella review has applied a formal strength-of-evidence framework to digital health education interventions for chronic disease self-management specifically in this population. The aim of this umbrella review was to determine whether sufficient evidence supports the use of digital health education interventions for chronic disease self-management in community-dwelling adults aged 60 years or older. We also assessed the methodological quality of the included systematic reviews using A Measurement Tool to Assess Systematic Reviews 2 (AMSTAR 2), compared outcomes across populations and chronic disease clusters within the Population, Intervention, Comparison, Outcome framework, and provided recommendations for future evidence-based research.

## Methods

### Search strategy and selection criteria

This umbrella review was reported in accordance with the Preferred Reporting Items for Overviews of Reviews (PRIOR) statement (14) and the methodological framework proposed by Fusar-Poli and Radua (15). The review was prospectively registered in PROSPERO (CRD420261386392). Because this review synthesized previously published aggregate-level data and did not involve human participants, ethics approval was not required. We searched PubMed/MEDLINE, CINAHL Complete, the Cochrane Database of Systematic Reviews, Web of Science (Science Citation Index Expanded), and Scopus, with Epistemonikos searched as an additional source, for eligible reviews published between January 1, 2015, and the search date. Initial searches were conducted on May 1, 2026, in PubMed and the Cochrane Database of Systematic Reviews. Following internal review, additional search terms (telecare, counseling, self-monitoring, and self-assessment) were incorporated, and all databases were re-searched using the final search strategy on May 2, 2026. A simplified natural language search strategy was used for Epistemonikos. Searches were limited to publications in English or Turkish, and the reference lists of included reviews were manually screened for additional eligible studies. The complete search strategies for all databases are provided in Supplementary Table S1 (Supplementary Appendix, p.4). The study selection process is presented in Figure 1.

**Figure 1:**
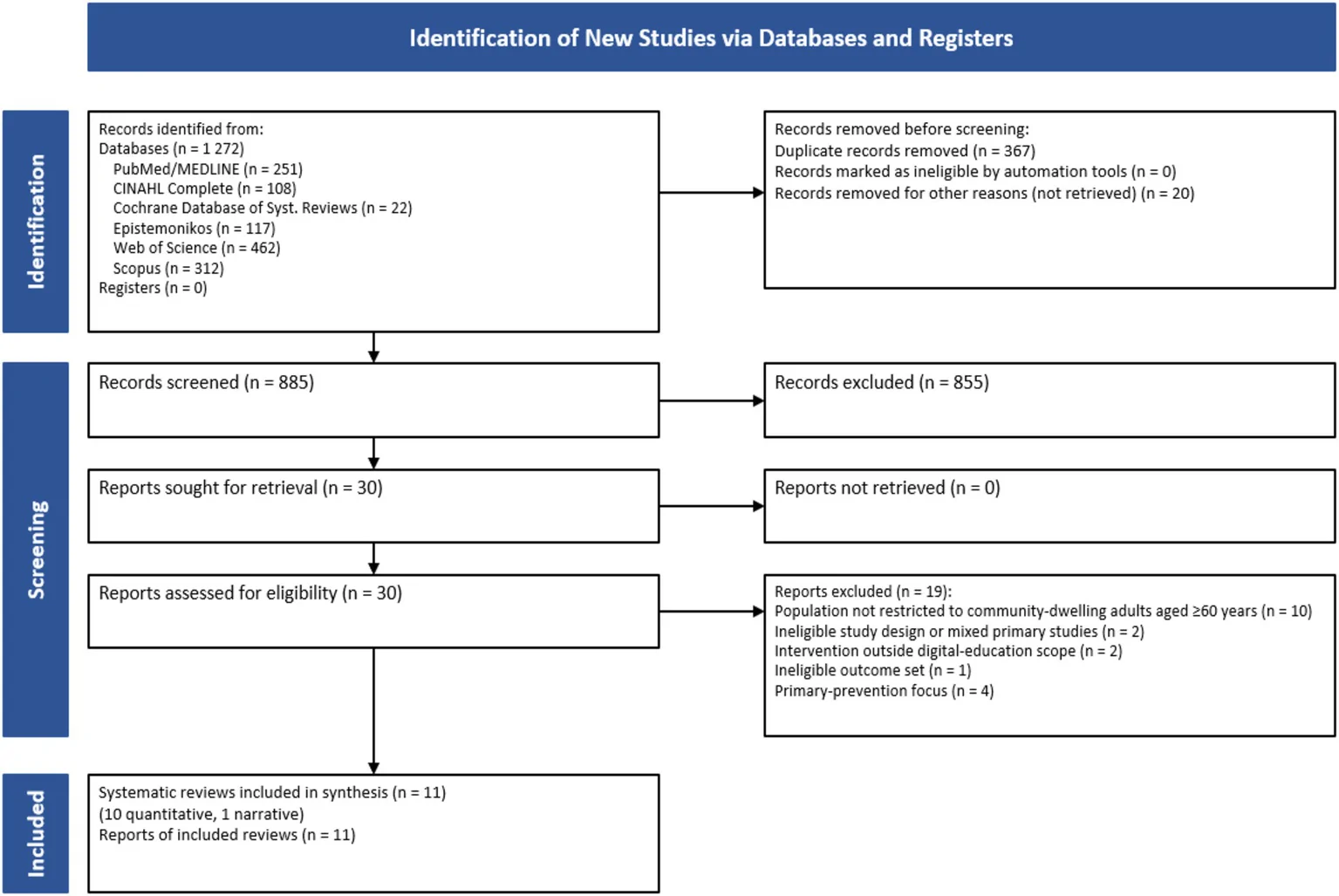
Preferred reporting items for systematic reviews and meta-analyses (PRISMA) 2020 flow diagram for study selection. Of the 30 full-text articles assessed for eligibility, 19 were excluded, including 10 because the synthesized population was not restricted to community-dwelling adults aged 60 years or older. Eleven systematic reviews were included in the umbrella review.

### Eligibility criteria

Eligibility criteria were defined according to the PICOS framework.

*Population:* Eligible populations included community-dwelling adults aged 60 years or older with one or more chronic noncommunicable diseases (NCDs), including type 2 diabetes, hypertension, heart failure, chronic obstructive pulmonary disease (COPD), coronary artery disease, chronic kidney disease, osteoarthritis, or multimorbidity. Eligibility was determined based on the mean age reported for the population contributing to the review-level estimate; reviews were eligible when the reported mean age was ≥60 years. Individual primary studies were not required to restrict enrollment exclusively to participants aged ≥60 years.

*Intervention:* Eligible interventions, delivered either alone or as the primary component of a multimodal program, included mobile health applications, telehealth and telemonitoring, wearable devices, web-based programs, text messaging, telecare, internet-based coaching, chatbot- or other artificial intelligence-based tools, and digital self-monitoring platforms incorporating an educational component.

*Comparator:* Eligible comparators included usual care, no intervention, attention control, nondigital education, or another digital intervention.

*Outcomes:* Reviews were required to report at least one health-related outcome relevant to chronic disease self-management, including disease-specific clinical indicators, health service use, self-management behaviors, self-efficacy, or health-related quality of life.

*Study design:* Systematic reviews with or without meta-analysis, including network meta-analyses, were eligible. Systematic reviews without meta-analysis contributed to the narrative synthesis only.

Exclusion criteria, we excluded reviews of institutionalized or hospitalized populations; reviews involving acute conditions, cancer, mental health disorders, dementia, or palliative care; interventions delivered exclusively in person, by telephone without digital technology, or solely to caregivers or health care professionals; reviews reporting only outcomes unrelated to chronic disease self-management; and reviews with a primary-prevention focus without a chronic disease management component.

After duplicate records were removed in Mendeley, two reviewers independently screened titles, abstracts, and full-text articles using Rayyan. Inter-rater agreement was assessed using Cohen’s *κ* for title and abstract screening, full-text screening, and methodological quality appraisal, with a predefined threshold of *κ* ≥ 0.80. For formal strength of evidence classification, only meta-analytic estimates derived from RCTs or other comparative study designs were eligible. Estimates from single-arm or pre-post studies were summarized descriptively, and the proportion of RCTs included in each systematic review was recorded.

### Outcomes

The primary outcomes included disease-specific clinical indicators, such as glycated hemoglobin, systolic and diastolic blood pressure, heart failure-related hospitalization or readmission, COPD exacerbation rate, and pulmonary function, as well as self-management behaviors and self-efficacy measured using validated instruments. Continuous outcomes were summarized as standardized mean differences or raw mean differences, whereas dichotomous and count outcomes were summarized as odds ratios, risk ratios, or hazard ratios. All effect estimates were standardized as equivalent Hedges’ g (eG) or equivalent odds ratios (eORs). For each outcome, data were extracted at the longest follow-up reported in each review. When available, estimates were extracted separately for short-term (<6 months), medium-term (6–12 months), and long-term (≥12 months) follow-up periods.

### Data extraction and quality assessment

Two reviewers independently assessed the methodological quality of the included systematic reviews using AMSTAR 2 (16). Each review was assigned an overall confidence rating (high, moderate, low, or critically low). These ratings informed the interpretation of the findings but were not used as exclusion criteria. For each primary study contributing to a meta-analytic outcome, we extracted the first author, publication year, effect estimate with its 95% confidence interval (CI), and the sample sizes of the intervention and control groups. We also extracted the total sample size, 95% prediction interval, *I*^2^ statistic, *p* value of the largest study, Egger’s test for small-study effects, and the excess significance bias (ESB) test required for evidence classification. Risk of bias due to missing results was also considered. The educational components of the interventions were extracted descriptively. However, educational dose could not be quantified because the included reviews did not consistently report standardized information on the frequency, duration, intensity, or theoretical basis of the educational components. Therefore, meta-regression according to educational intensity or theory-based design was not feasible. The characteristics of the included systematic reviews and the digital health education interventions are summarized in Tables 1, 2.

**Table 1:** Characteristics of the 11 included systematic reviews.

Review	Disease cluster	Contribution	RCTs (*k*)	AMSTAR 2	Highest-class outcome
Son 2020 (36)	Heart failure	Quantitative	4	Critically low	Class IV (length of stay)
Allida 2020 (37)	Heart failure	Quantitative	5	Low	Not significant
Wong 2022 (24)	Multimorbidity	Quantitative	13	Low	Class IV (self-efficacy and quality of life)
Yang 2022 (38)	Diabetes	Quantitative	9	Critically low	Not significant (glycated hemoglobin)
Lee 2023 (26)	Type 2 diabetes mellitus	Quantitative	9	Critically low	Class IV (postprandial glucose and triglycerides)
Zhang 2024 (18)	Heart failure	Quantitative	13	High	Class IV (medication adherence)
Park 2025 (25)	Multimorbidity	Quantitative	9	Critically low	Class IV (physical activity)
Wang 2025 (22)	COPD	Quantitative	26	High	Class IV [dyspnea and EuroQol 5-Dimension (EQ-5D)]
Tam 2022 (23)	Hypertension	Quantitative	6	Critically low	Class IV (systolic blood pressure and medication adherence)
Ni 2024 (32)	Heart failure	Quantitative	15	High	Class IV (heart failure-related hospitalization)
Lippi 2023 (21)	COPD	Narrative	—	High	—

*k*, number of primary randomized controlled trials (RCTs) contributing to the review’s meta-analyses. The highest-class outcome represents the highest Ioannidis evidence classification achieved among the outcomes within each review; no review reached Class I, II, or III evidence. EQ-5D = EuroQol 5-Dimension.

**Table 2:** Digital health education intervention modalities across the 11 included systematic reviews.

Review	Disease focus	Primary digital modality	Delivery channel	Educational component
Son 2020	Heart failure	Telemonitoring	Home monitoring devices with remote follow-up	Vital sign monitoring with educational feedback
Allida 2020	Heart failure	Self-management programs	Mobile applications, telephone, and web-based platforms	Heart failure education and symptom monitoring
Wong 2022	Multimorbidity	Digital self-management platforms	Web-based and mobile platforms	Self-management education for multiple chronic conditions
Yang 2022	Diabetes	Mixed digital education	Web portals, short message service (SMS), and mobile applications	Diabetes self-management education
Lee 2023	Type 2 diabetes mellitus	Smartphone-based interventions	Mobile applications	Self-monitoring with educational prompts
Zhang 2024	Heart failure	Mobile applications	Smartphone applications	Medication reminders and self-care education
Park 2025	Multimorbidity	Digital self-care interventions	Mobile applications and telehealth	Self-management education for multiple chronic conditions with mental health components
Wang 2025	Chronic obstructive pulmonary disease (COPD)	Digital pulmonary rehabilitation	Mobile applications, telerehabilitation, and wearable devices	Disease education, exercise training, and symptom monitoring
Tam 2022	Hypertension	Mobile application-based interventions	Smartphone applications with blood pressure (BP) monitoring	Hypertension self-management education
Ni 2024	Heart failure	Multi-component mobile health (mHealth) interventions	Mobile applications, web-based platforms, and wearable devices	Heart failure education and self-monitoring
Lippi 2023	COPD	Mixed digital interventions	Multiple delivery channels	Narrative review of digital interventions for COPD

BP, blood pressure; COPD, chronic obstructive pulmonary disease; mHealth, mobile health; SMS, short message service. (Quality of life was not used in this table and has therefore been omitted from the abbreviations).

### Statistical analysis

All statistical analyses were performed in R (version 4.5 or later) using the metaumbrella package (17). For each meta-analytic outcome, we re-estimated the pooled effect using a random-effects model. The registered protocol prespecified the DerSimonian–Laird estimator with the Hartung–Knapp adjustment; however, we used the restricted maximum likelihood (REML) estimator, which is the default in the metaumbrella package and is generally preferred for estimating between-study variance. Because REML is less conservative than the protocol-specified Hartung–Knapp approach, the absence of any Class I–III evidence cannot be attributed to the use of an overly conservative estimator. Effect estimates were standardized as equivalent Hedges’ g (eG) or equivalent odds ratios (eORs). Before pooling, we screened confidence intervals (CIs) for log-scale asymmetry. Two risk ratio estimates reported by Zhang 2024 (18) exceeded the predefined 10% log-asymmetry threshold (37.6 and 11.7%) and were reconstructed using log-symmetric methods before analysis. The strength of evidence was graded according to the Ioannidis criteria.16 Evidence was classified as Class I (convincing) when the meta-analysis included more than 1,000 cases, *p* < 1 × 10^−6^, *I*^2^ < 50%, a 95% CI excluding the null, no evidence of small-study effects or excess significance bias (ESB), and a statistically significant effect in the largest study. Class II (highly suggestive) required more than 1,000 cases, *p* < 1 × 10^−6^, and a significant effect in the largest study. Class III (suggestive) required more than 1,000 cases and *p* < 1 × 10^−3^. Class IV (weak) required *p* < 0.05, whereas findings with *p* ≥ 0.05 were classified as non-significant. For findings reaching Class I or II evidence, a manual assessment using the Grading of Recommendations Assessment, Development and Evaluation (GRADE) approach with GRADEpro GDT (19) was prespecified as a second-tier evaluation. When two or more reviews reported the same outcome, we prioritized the most recent review with the highest AMSTAR 2 rating and performed sensitivity analyses using alternative reviews. Overlap among primary studies was quantified using the CCA calculated with the Pieper formula (20). CCA values were interpreted according to Pieper et al. as 0–5% (slight), 6–10% (moderate), 11–15% (high), and >15% (very high). For classification purposes, decimal CCA values were rounded to the nearest whole percentage before applying these categories.

## Results

### Study selection and characteristics

We identified 1,272 records across six databases: PubMed/MEDLINE (*n* = 251), CINAHL Complete (*n* = 108), the Cochrane Database of Systematic Reviews (*n* = 22), Epistemonikos (*n* = 117), Web of Science (*n* = 462), and Scopus (*n* = 312). After removing 367 duplicate records and 20 records that could not be retrieved, 885 records underwent title and abstract screening. Of these, 855 were excluded, primarily because of an ineligible population (*n* = 361), ineligible intervention (*n* = 326), or ineligible publication type (*n* = 113). We assessed 30 full-text articles for eligibility, of which 19 were excluded for the following reasons: the synthesized population was not restricted to community-dwelling adults aged 60 years or older (*n* = 10), ineligible study design or inclusion of mixed primary study designs (*n* = 2), interventions outside the scope of digital health education (*n* = 2), ineligible outcome measures (*n* = 1), or a primary prevention focus without a chronic disease management component (*n* = 4). Ultimately, 11 systematic reviews met the eligibility criteria (Figure 1). Of the included reviews, 10 provided quantitative meta-analytic data suitable for re-analysis, whereas one review (Lippi 2023) (21) was included only in the narrative synthesis. Across the 10 quantitative reviews, we extracted data from 199 primary RCTs contributing to 44 distinct meta-analytic outcomes across five chronic disease clusters: diabetes mellitus (five outcomes from two reviews), heart failure (17 outcomes from four reviews), COPD (eight outcomes from one review [Wang 2025] (22)), hypertension (four outcomes from one review [Tam 2022] (23)), and multimorbidity (10 outcomes from two reviews [Wong 2022] (24) and [Park 2025] (25)). All meta-analytic estimates included in the formal evidence classification were derived from RCTs. AMSTAR 2, three of the 10 quantitative reviews were rated as high confidence, two as low confidence, and five as critically low confidence.

### Strength of evidence

Across the 44 pooled outcomes, none met the criteria for convincing (Class I), highly suggestive (Class II), or suggestive (Class III) evidence. Fifteen outcomes (34.1%) were classified as weak (Class IV), whereas 29 (65.9%) were non-significant (pooled *p* ≥ 0.05). Because no outcomes met the criteria for Class I or II evidence, the prespecified second-tier GRADE assessment was not conducted. The complete absence of Class I–III evidence, despite 15 nominally significant pooled effects, indicates that when the evidence base is restricted to community-dwelling adults aged 60 years or older, no digital health education intervention for chronic disease self-management is currently supported by high-credibility evidence at the umbrella review level. The proportion of non-significant outcomes in the present review (65.9%) was higher than the 42% reported by Sun et al. (13) in their umbrella review of mobile phone-based interventions for chronic diseases across all age groups. That review applied the same Fusar-Poli–Radua (15) framework and identified convincing (Class I) evidence for reductions in glycated hemoglobin among individuals with type 2 diabetes. The distribution of evidence classes by chronic disease cluster is presented in Table 3, and the complete classification of all 44 outcomes is shown in Figure 2.

**Table 3:** Distribution of ioannidis evidence classes across the 44 meta-analytic outcomes by chronic disease cluster.

Disease cluster	Outcomes (*k*)	N, range (median)	Class I	Class II	Class III	Class IV	Not significant	Contributing reviews
Diabetes mellitus	5	273–4,264 (1,793)	0	0	0	2	3	Lee 2023; Yang 2022
Heart failure	17	29–2,127 (942)	0	0	0	5	12	Allida 2020; Son 2020; Zhang 2024; Ni 2024
Chronic obstructive pulmonary disease (COPD)	8	265–1,603 (510)	0	0	0	3	5	Wang 2025
Hypertension	4	542–1,103 (898)	0	0	0	2	2	Tam 2022
Multimorbidity	10	406–2,929 (1,416)	0	0	0	3	7	Wong 2022; Park 2025
Total	44	29–4,264 (1,080)	0 (0%)	0 (0%)	0 (0%)	15 (34.1%)	29 (65.9%)	10 quantitatively contributing reviews

**Figure 2:**
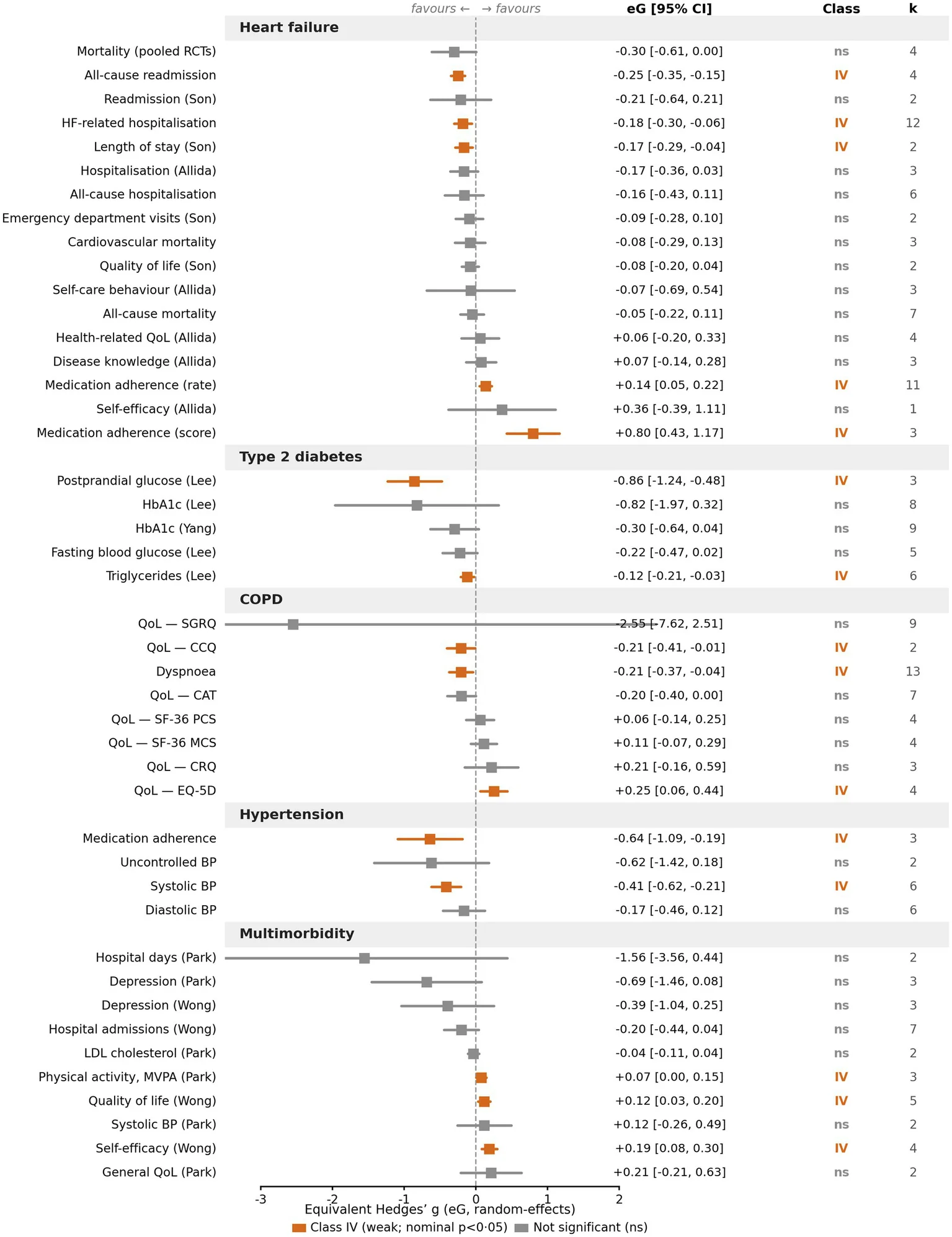
Evidence map of the 44 meta-analytic outcomes by chronic disease cluster. Pooled equivalent Hedges’ g (eG) values with 95% confidence intervals (CIs), the Ioannidis evidence classification, and the number of contributing primary randomized controlled trials (RCTs) (*k*) are shown for each outcome. No outcome met the criteria for Class I, II, or III evidence.

### Weak and non-significant evidence

Of the 44 pooled outcomes, 15 were statistically significant but did not meet the criteria for higher evidence classes and were therefore classified as Class IV (weak evidence). The largest effect sizes were observed for medication adherence in heart failure (equivalent Hedges’ g [eG] = 0.797, 95% CI = 0.427 to 1.168; Zhang 2024 (18)), reductions in postprandial glucose among individuals with type 2 diabetes (eG = −0.857, 95% CI − 1.235 to −0.478; Lee 2023 (26); *p* = 9.36 × 10^−6^), reductions in systolic blood pressure among individuals with hypertension (eG = −0.415, 95% CI − 0.620 to −0.210; Tam 2022 (23); *p* = 7.32 × 10^−5^), and reductions in all-cause hospital readmission among individuals with heart failure (eG = −0.250, 95% CI − 0.349 to −0.151; *p* = 7.37 × 10^−7^). However, all of these outcomes included relatively few primary studies (*k* = 2–13) and total participant numbers below the Ioannidis thresholds for Class III evidence or had 95% prediction intervals that crossed the null. An additional 29 outcomes were not statistically significant. Disease cluster-specific forest plots for diabetes mellitus and heart failure are presented in Figure 3.

**Figure 3:**
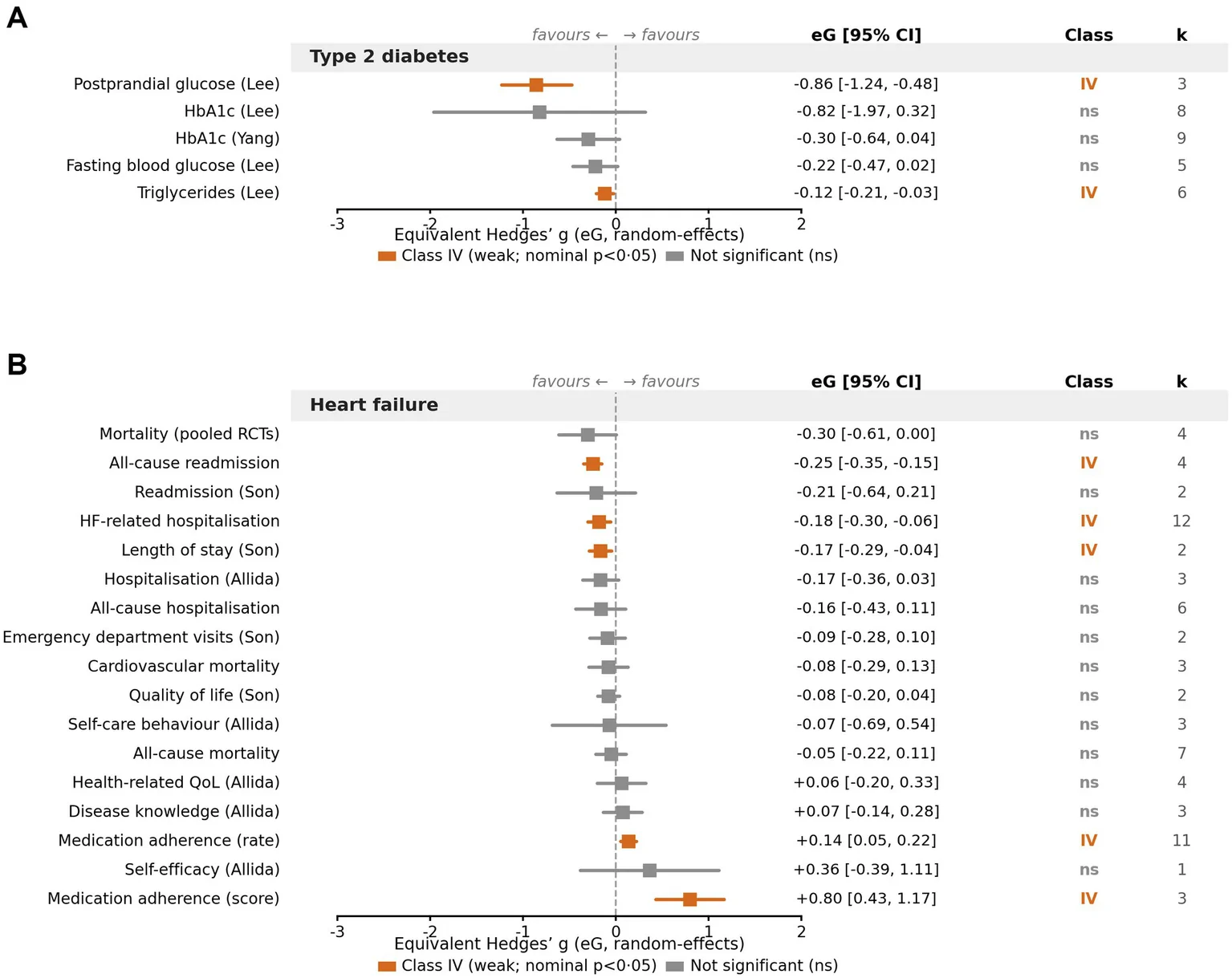
Cluster-specific forest plots. **(A)** Diabetes mellitus (five outcomes) and **(B)** heart failure (17 outcomes). Pooled equivalent Hedges’ g (eG) values with 95% confidence intervals (CIs) are presented for each outcome. Outcomes classified as Class IV (weak evidence) are distinguished from non-significant outcomes.

### Cross-review overlap and independence of the evidence

Across the 10 quantitatively contributing reviews, we identified 102 unique primary RCTs from 109 citation occurrences. The overall CCA was 0.76%, well below the 5% threshold for slight overlap, indicating that the pooled estimates were derived from largely independent sets of primary studies. The CCA values stratified by chronic disease cluster were 2.94% for heart failure and 4.76% for multimorbidity, both of which fell within the slight overlap range. The CCA was 5.88% (rounded to 6% for classification), indicating moderate overlap. The COPD and hypertension clusters were each represented by a single systematic review; therefore, overlap could not be assessed for these disease clusters. Six primary RCTs were included in two or more systematic reviews, and one trial appeared in three reviews. No primary study was included in four or more reviews. The complete pairwise overlap matrix is presented in Figure 4.

**Figure 4:**
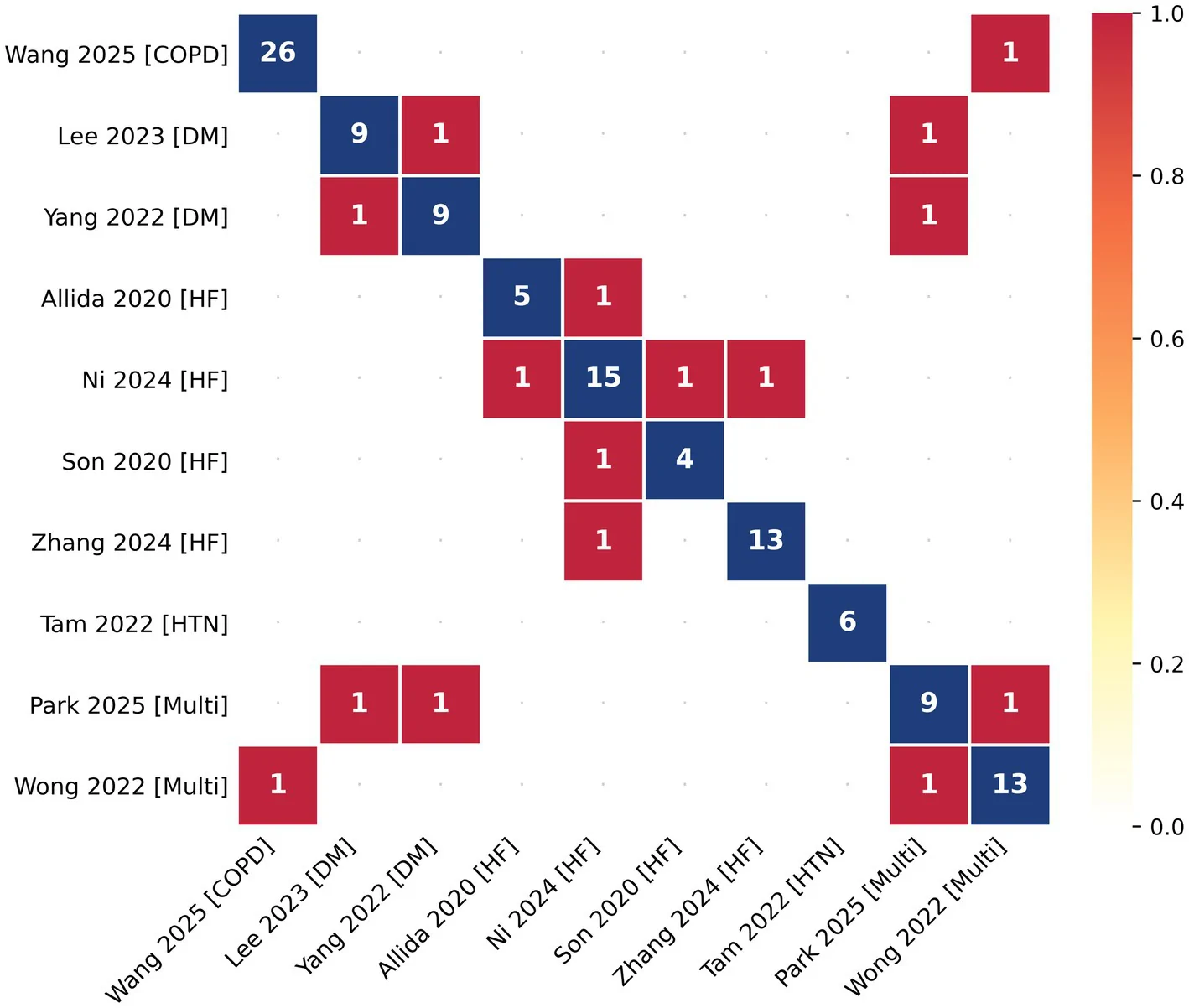
Heatmap of the corrected covered area (CCA) across the included systematic reviews. Diagonal cells (navy) indicate the number of primary randomized controlled trials (RCTs) included in each review, whereas off-diagonal cells (red) indicate the number of primary RCTs shared between each pair of reviews. The overall CCA was 0.76%, indicating slight overlap (<5%) among the included reviews.

### Heterogeneity, prediction intervals, and statistical power

Between-study heterogeneity varied substantially across outcomes, with *I*^2^ values ranging from 0 to 99.3%. High heterogeneity (*I*^2^ > 75%) was observed in 14 outcomes and was distributed across the heart failure, multimorbidity, hypertension, and diabetes mellitus clusters rather than being concentrated within a single disease cluster. The 95% prediction interval included the null value for all 44 outcomes, indicating that none demonstrated evidence of a consistent effect across future comparable studies. Total sample sizes ranged from 29 to 4,264 participants per outcome (median 1,080), with cluster-specific ranges of 29–2,127 for heart failure, 273–4,264 for type 2 diabetes mellitus, 265–1,603 for COPD, 542–1,103 for hypertension, and 406–2,929 for multimorbidity (Table 3; Supplementary Table S4). Forty-three of the 44 outcomes had total sample sizes above 200 participants; the only exception was self-efficacy in heart failure (Allida 2020), which was based on a single trial (N = 29).

## Discussion

In this umbrella review of digital health education interventions for chronic disease self-management among community-dwelling adults aged 60 years or older, we synthesized 11 systematic reviews and reanalyzed 44 meta-analytic outcomes across five chronic disease clusters. Although the evidence base included data from 199 primary RCTs and demonstrated minimal overlap among reviews (overall CCA was 0.76%), no outcome met the criteria for convincing (Class I), highly suggestive (Class II), or suggestive (Class III) evidence. Only 15 of the 44 outcomes (34.1%) were classified as weak (Class IV), whereas 29 (65.9%) were not statistically significant. Because no outcome achieved Class I or II evidence, the prespecified GRADE assessment was not performed. Overall, these findings indicate that, when the evidence is restricted to community-dwelling older adults, digital health education interventions for chronic disease self-management are currently supported only by low-certainty evidence at the umbrella review level rather than by high-credibility evidence.

The proportion of non-significant outcomes in the present review (65.9%) was substantially higher than the 42% reported by Sun et al. (13) in their umbrella review of mobile phone-based interventions for chronic diseases across all age groups. That review applied the same Fusar-Poli–Radua framework (15) and identified convincing (Class I) evidence for reductions in glycated hemoglobin among individuals with type 2 diabetes.

The divergence between these findings is informative. The single Class I finding reported in the broader literature was no longer evident when the evidence base was restricted to community-dwelling older adults. This suggests that intervention effects observed in mixed-age populations cannot be assumed to generalize to older adults, whose clinical, functional, and technological needs differ substantially. Age-related barriers—including declines in cognitive and physical function, sensory impairments, and reduced motivation—may limit the usability of digital health technologies and reduce sustained engagement, thereby diminishing intervention effectiveness (8). Because digital health education interventions depend on active participation, behavior change, and long-term self-management rather than technology use alone, these age-related barriers may have a disproportionate impact on intervention outcomes among older adults (27). This interpretation is supported by previous systematic reviews. Health information technology-based diabetes self-management education produced only modest improvements in glycemic outcomes among medically underserved adults, with benefits diminishing after 12 months (28). Similarly, mobile health (mHealth) interventions in adults older than 50 years demonstrated beneficial but inconsistent effects that varied according to intervention modality (29). In addition, app-based interventions designed to improve medication adherence in individuals with type 2 diabetes yielded statistically significant benefits in only a minority of trials (30, 31). A further consideration is electronic health (eHealth) literacy, defined by Norman and Skinner as the ability to seek, understand, and appraise health information from electronic sources and to apply it to a health problem (27). eHealth literacy is plausibly not merely a barrier to access but a key effect modifier: an older adult who cannot navigate an application or interpret digitally delivered information is unlikely to benefit even from educational content of high quality. This distinction separates intervention effectiveness—whether the educational content works when it is successfully used—from implementation feasibility—whether older users can access and operate the technology in the first place. Because baseline eHealth literacy varies widely in this population and was rarely measured or reported in the underlying trials, its moderating role could not be examined at the umbrella level. Future randomized controlled trials should measure baseline eHealth literacy with validated instruments and consider stratified randomization by literacy level, so that efficacy among capable users can be distinguished from failures of engagement and access; this would also help identify the digital-literacy support (e.g., structured onboarding, simplified interfaces, or caregiver assistance) required for these interventions to succeed in routine care.

These access-related considerations also bear on global health equity. Although more than 80% of adults aged 60 years or older are projected to live in low- and middle-income countries (LMICs) by 2050 (1), the reviews synthesized here were drawn overwhelmingly from high-income settings, where connectivity, device ownership, and technical support are comparatively assured. The current estimates therefore describe digital health education under favorable implementation conditions and cannot be assumed to transfer to LMICs, where greater infrastructural and digital-literacy barriers may render the same interventions less effective, or infeasible as delivered. Because the populations expected to bear the greatest future burden of chronic disease in later life are precisely those least represented in the evidence, uncritical extrapolation risks widening inequities; advancing equity will require adequately powered trials in these settings that explicitly address barriers to digital access (2).

The complete absence of Class I–III evidence cannot be explained by insufficient statistical power or an overly conservative analytical approach. Statistical power to detect a moderate effect was consistently high for outcomes including more than 200 participants, which represented the predominant pattern in our dataset. In addition, all pooled effects were re-estimated using a REML random-effects model, which is less conservative than the Hartung–Knapp adjustment prespecified in our protocol. Therefore, the lack of high-credibility evidence is more likely attributable to limitations of the underlying evidence base than to the analytical methods used. Three characteristics of the available evidence are particularly noteworthy. First, most outcomes were based on relatively few primary trials (*k* = 2–13) and total sample sizes below the Ioannidis thresholds required for higher evidence classifications. Second, the 95% prediction interval included the null value for all 44 outcomes, indicating that future studies could plausibly demonstrate no intervention benefit even when the pooled effect estimate was statistically significant. This finding suggests limited robustness and poor generalizability of the observed effects across different clinical settings. Third, between-study heterogeneity was frequently substantial (*I*^2^ up to 99.3%), with 14 outcomes exceeding 75%. This heterogeneity was distributed across disease clusters rather than concentrated within a single clinical area, suggesting that it primarily reflected differences in intervention characteristics–including modality, intensity, educational content, study populations, and comparators interventions–rather than disease-specific factors. Given the diversity of digital health education interventions, this degree of heterogeneity is expected and likely contributed to the inconsistency of the observed findings. The largest Class IV (weak evidence) effects included improvements in medication adherence among individuals with heart failure (equivalent Hedges’ g [eG] = 0.797) (18), reductions in postprandial glucose among individuals with type 2 diabetes (eG = −0.857) (26), reductions in systolic blood pressure among individuals with hypertension (eG = −0.415) (23), and lower all-cause hospital readmission among individuals with heart failure (eG = −0.250). Although these findings are clinically meaningful and warrant further investigation in adequately powered trials, the current evidence is insufficient to consider them definitive rather than exploratory.

The credibility of the umbrella review findings is further limited by the methodological quality of the contributing systematic reviews. Of the 10 reviews that provided quantitative data, only three were rated as high confidence according to AMSTAR 2, whereas two were rated as low confidence and five as critically low confidence (16). Notably, even the highest-quality reviews achieved no stronger than Class IV evidence for their most robust outcomes. This observation suggests that the primary limitation lies not only in the quality of the systematic reviews but also in the maturity of the underlying evidence base. Many of the included RCTs were small, had short follow-up periods, and reported outcomes inconsistently. The minimal overlap among systematic reviews (CCA = 0.76%) further indicates that these limitations reflect a broad and largely independent body of evidence rather than repeated inclusion of the same primary studies. This strengthens confidence that the overall weakness of the evidence is an inherent characteristic of the field rather than an artifact of review overlap. Advancing the evidence base will require not only additional well-designed RCTs but also improvements in methodological rigor, longer follow-up periods, standardized outcome reporting, and high-quality systematic reviews conducted in accordance with AMSTAR 2 standards. Such improvements are essential for generating high-credibility evidence in future umbrella reviews.

These findings have important implications for nursing practice and community-based care. Several of the included systematic reviews evaluated nurse-led or nursing-relevant interventions, including nurse-led telehealth programs that promote self-care (24), text message-based interventions for hypertension management (23), mobile application-based interventions for heart failure (32), and digital pulmonary rehabilitation in COPD (22). Collectively, these findings underscore the central role of public health and community nurses in implementing digitally supported self-management education. Although the overall certainty of evidence was low, the variability in intervention effects suggests that effectiveness among older adults may depend less on the specific digital modality than on how well the intervention is tailored to an individual’s functional capacity, preferences, and evolving health needs. Interventions that adapt to users’ changing needs, rather than relying on a one-size-fits-all approach, represent a promising direction for future development (33, 34), Similarly, digital tools that support frailty monitoring and healthy aging in community settings may offer additional benefits (35). However, the current evidence does not support the widespread adoption of any single digital modality for chronic disease self-management in community-dwelling older adults. Nurses are uniquely positioned to implement digital health education interventions selectively, address age-related barriers to technology engagement, and incorporate structured outcome evaluation into routine practice. These efforts can simultaneously improve patient care and contribute to strengthening the evidence base needed to guide future implementation.

## Strengths and limitations

This umbrella review has several notable strengths. It synthesized evidence across five chronic disease clusters and a broad range of clinical, behavioral, and self-efficacy outcomes, providing a comprehensive assessment of digital health education interventions for chronic disease self-management among community-dwelling older adults. In addition, it applied a formal and reproducible strength-of-evidence framework, quantified overlap among primary studies to confirm a largely independent evidence base (CCA = 0.76%), and reanalyzed all pooled estimates using a robust random-effects model. Together, these features indicate that the absence of high-credibility evidence reflects the underlying evidence base rather than the analytical approach.

Despite these strengths, the limitations of this umbrella review and gaps in the underlying literature on meta-analyses and randomized controlled trials (RCTs) should be considered. First, as an umbrella review, our conclusions are limited by the availability and quality of existing systematic reviews and meta-analyses. Only 11 reviews met the eligibility criteria, and the maturity of the evidence varied across chronic disease clusters. COPD and hypertension were each represented by a single systematic review, and several outcomes were based on relatively few primary trials (*k* = 2–13) with total sample sizes below the Ioannidis thresholds required for higher evidence classifications. Consequently, these interventions should be re-evaluated as larger RCTs and updated meta-analyses focused specifically on community-dwelling older adults become available.

Second, our eligibility criteria were intentionally restrictive. We limited the population to community-dwelling adults aged 60 years or older and excluded institutionalized and hospitalized populations; studies involving acute conditions, cancer, mental health disorders, dementia, or palliative care; and interventions delivered exclusively in person, by telephone without digital technology, or solely to caregivers or health care professionals. Although these criteria strengthened the internal validity of the review, they reduced the generalizability of the findings. In addition, limiting inclusion to systematic reviews published in English or Turkish may have resulted in the omission of relevant evidence published in other languages. Relatedly, eligibility was based on a reported mean age of ≥60 years rather than requiring all participants in the underlying primary studies to be aged ≥60 years; consequently, some included reviews may have contained primary studies with mixed-age samples, which may limit the age-specific applicability of the findings.

Third, we graded the strength of evidence using the Fusar-Poli–Radua application of the Ioannidis criteria (15), which classifies evidence on the basis of statistical parameters, including sample size, *p* values, heterogeneity, prediction intervals, small-study effects, and ESB. However, this framework does not directly account for the risk of bias of the primary RCTs or incorporate an optimal information size criterion. We addressed this limitation only in part by independently assessing the methodological quality of the included systematic reviews using AMSTAR 2 (16).

Fourth, our re-analysis was constrained by the outcome definitions, effect measures, and reporting quality of the included systematic reviews. When standard deviations or other statistics required for effect size conversion were unavailable, some estimates could not be standardized. In addition, one systematic review contributed only narrative findings and therefore could not be included in the quantitative re-analysis.

Fifth, and relatedly, formal assessment of small-study effects and publication bias has limited statistical power when few trials are available and between-study heterogeneity is substantial, as was frequently observed in this review. Consequently, the certainty of some findings may have been either overestimated or underestimated. In addition, few systematic reviews evaluated potential moderators of intervention effectiveness, preventing us from examining how factors such as intervention theory, intervention dose, delivery channel, health care professional involvement, or the educational component influenced outcomes. These factors are plausible contributors to the substantial between-study heterogeneity observed across the included studies (*I*^2^ up to 99.3%). Moreover, heterogeneity and inconsistent reporting of the frequency, duration, intensity, and theoretical basis of the educational components precluded quantification of educational dose and any assessment of whether intervention intensity or theory-based design influenced effect estimates.

Finally, important gaps remain in the primary RCT evidence. Studies conducted in low- and middle-income countries and the Global South are underrepresented, despite projections indicating most older adults will reside in these regions in the coming decades. Follow-up periods were generally short, limiting assessment of the long-term sustainability of intervention effects and highlighting the need for trials with follow-up periods of at least 12 months. In addition, adverse events were infrequently reported, restricting the current understanding of the safety profile of digital health education interventions for chronic disease self-management among older adults.

## Conclusion

In this umbrella review restricted to community-dwelling adults aged 60 years or older, in which 11 systematic reviews and 44 meta-analytic outcomes across five chronic disease clusters were synthesized and reanalyzed, the current umbrella-level evidence does not demonstrate that digital health education interventions improve chronic disease self-management among community-dwelling older adults. None of the 44 outcomes reached convincing (Class I), highly suggestive (Class II), or suggestive (Class III) evidence; 15 (34.1%) were graded as weak (Class IV) and 29 (65.9%) were non-significant. This absence of high-credibility evidence should be interpreted as reflecting an immature and heterogeneous evidence base—characterized by few trials, short follow-up, and low methodological quality—rather than as evidence that these interventions are ineffective. Several self-management outcomes, including medication adherence in heart failure, glycemic outcomes in type 2 diabetes, and blood pressure control in hypertension, showed weak but clinically plausible signals that warrant confirmation. Until adequately powered randomized controlled trials with longer follow-up and standardized outcomes, together with higher-quality systematic reviews focused specifically on older adults, become available, digital health education should be offered selectively and as a complement to—rather than a replacement for—established models of chronic disease care, tailored to the functional capacity, digital literacy, and preferences of older adults.

## Data Availability

The original contributions presented in the study are included in the article/Supplementary material, further inquiries can be directed to the corresponding author.
