# Supplementary material for: Effectiveness of digital health education interventions for chronic disease self-management in community-dwelling older adults: an umbrella review and evidence classification
Source: Front Public Health. 2026 Oct 12;14:1948010. doi: 10.3389/fpubh.2026.1948010 (PMC13696277; doi:10.3389/fpubh.2026.1948010)
Supplement: Supplementary file 1 [file Data_sheet_1.docx]

**Supplementary Appendix**

**Effectiveness of digital health education interventions for chronic disease self-management in community-dwelling older adults: an umbrella review and evidence classification**

Supplement to the main manuscript. This appendix provides the full search strategies, reporting checklists, and supplementary tables and figures referenced in the main text. PROSPERO registration: CRD420261386392.

## **Contents**

[**Contents**	1](#_Toc234912901)

[**1. Supplementary methods**	2](#_Toc234912902)

[1.1 Search strategy and information sources	2](#_Toc234912903)

[1.2 Screening and inter-rater agreement	2](#_Toc234912904)

[1.3 Evidence classification criteria (Ioannidis / Fusar-Poli–Radua)	2](#_Toc234912905)

[1.4 Corrected covered area (CCA)	2](#_Toc234912906)

[**2. PRISMA 2020 checklist**	2](#_Toc234912907)

[**3. Supplementary tables**	4](#_Toc234912908)

[Table S1. Full electronic search strategy, by database	4](#_Toc234912909)

[Table S2. Studies excluded at full-text screening, with reasons (n = 19)	5](#_Toc234912910)

[Table S3. AMSTAR 2 item-level appraisal of the included systematic reviews	7](#_Toc234912911)

[Table S4. Full evidence classification of all 44 meta-analytic outcomes	8](#_Toc234912912)

[Table S5. Primary-study overlap underlying the corrected covered area (CCA)	10](#_Toc234912913)

[Table S6. Corrected covered area (CCA), overall and by disease cluster	10](#_Toc234912914)

**1. Supplementary methods**

**1.1 Search strategy and information sources**

We searched six bibliographic databases for systematic reviews, with or without meta-analysis, published between January 1, 2015, and May 2, 2026: PubMed/MEDLINE, CINAHL Complete, the Cochrane Database of Systematic Reviews, Web of Science (Science Citation Index Expanded), Scopus, and Epistemonikos. Initial searches were run on May 1, 2026, in PubMed and the Cochrane Database of Systematic Reviews; following internal review, additional terms (telecare, counseling, self-monitoring, self-assessment) were incorporated and all databases were re-searched using the final strategy on May 2, 2026. A simplified natural-language strategy was used for Epistemonikos. Searches were limited to publications in English or Turkish, and the reference lists of included reviews were screened manually for additional eligible studies. The full, line-by-line search strategy for each database is provided in Table S1.

**1.2 Screening and inter-rater agreement**

Duplicate records were removed in Mendeley. Two reviewers independently screened titles, abstracts, and full texts using Rayyan. Inter-rater agreement was assessed with Cohen’s κ for title/abstract screening, full-text screening, and methodological quality appraisal, against a predefined threshold of κ ≥ 0.80. Disagreements were resolved by discussion and, where necessary, adjudication by a third reviewer.

**1.3 Evidence classification criteria (Ioannidis / Fusar-Poli–Radua)**

For each meta-analytic outcome, the pooled effect was re-estimated under a random-effects model with restricted maximum likelihood (REML) estimation in the metaumbrella R package (version 4.5 or later). Effects were standardized as equivalent Hedges’ g (eG) or equivalent odds ratios (eOR). The strength of evidence was graded against the Ioannidis criteria as operationalized by Fusar-Poli and Radua:

| **Class** | **Criteria (all must be met)** |
| --- | --- |
| I — Convincing | >1,000 cases; p < 1 × 10⁻⁶; I² < 50%; 95% CI excluding the null; no small-study effects; no excess significance bias (ESB); significant effect in the largest study. |
| II — Highly suggestive | >1,000 cases; p < 1 × 10⁻⁶; significant effect in the largest study. |
| III — Suggestive | >1,000 cases; p < 1 × 10⁻³. |
| IV — Weak | p < 0.05 (not meeting higher-class criteria). |
| Non-significant | p ≥ 0.05. |

For each contributing outcome we extracted the total number of cases, the 95% prediction interval, I², the p value of the largest study, Egger’s test for small-study effects, and the excess significance bias (ESB) test. For any outcome reaching Class I or II, a manual GRADE assessment (GRADEpro GDT) was prespecified as a second-tier evaluation; because no outcome reached Class I or II, GRADE was not performed.

**Protocol deviation:** the registered protocol prespecified the DerSimonian–Laird estimator with the Hartung–Knapp adjustment; the REML estimator (the metaumbrella default) was used instead. Because REML is less conservative than Hartung–Knapp, the absence of Class I–III evidence cannot be attributed to an overly conservative estimator. Two risk-ratio estimates from Zhang 2024 exceeded the predefined 10% log-asymmetry threshold (37.6% and 11.7%) and were reconstructed using log-symmetric methods before pooling.

## **1.4 Corrected covered area (CCA)**

Overlap among primary studies was quantified using the corrected covered area (CCA) with the Pieper formula. CCA was interpreted using established thresholds: <5%, slight overlap; 6–10%, moderate; 11–15%, high; >15%, very high. CCA was calculated for the overall evidence base and separately for each disease cluster represented by at least two systematic reviews. The primary-study citation matrix underlying these calculations is provided in Table S5, and cluster-level values in Table S6.

**2. PRISMA 2020 checklist**

Completed PRISMA 2020 checklist. Because this is an overview of reviews, reporting also followed the PRIOR statement (Preferred Reporting Items for Overviews of Reviews); the completed PRIOR checklist should be attached as Section 2b using the official form from the EQUATOR Network.

| **Section** | **#** | **Checklist item** | **Location** |
| --- | --- | --- | --- |
| **TITLE** |  |  |  |
| Title | 1 | Identify the report as a systematic review. | Title page |
| **ABSTRACT** |  |  |  |
| Abstract | 2 | See the PRISMA 2020 for Abstracts checklist. | Summary |
| **INTRODUCTION** |  |  |  |
| Rationale | 3 | Describe the rationale in the context of existing knowledge. | Introduction |
| Objectives | 4 | Provide an explicit statement of the objective(s)/question(s). | Introduction (final paragraph) |
| **METHODS** |  |  |  |
| Eligibility criteria | 5 | Specify inclusion/exclusion criteria and how studies were grouped. | Methods – Search strategy and selection criteria |
| Information sources | 6 | Specify all databases/sources and the date last searched. | Methods; Suppl. Methods 1.1 |
| Search strategy | 7 | Present the full search strategy for all databases. | Table S1 |
| Selection process | 8 | Specify methods to decide inclusion, incl. reviewers and automation. | Methods; Suppl. Methods 1.2 |
| Data collection process | 9 | Specify methods of data collection, incl. reviewers and automation. | Methods – Data extraction and quality assessment |
| Data items | 10a | List and define all outcomes for which data were sought. | Methods – Outcomes |
| Data items | 10b | List and define all other variables for which data were sought. | Methods – Data extraction |
| Study risk of bias | 11 | Specify methods to assess risk of bias in included studies. | Methods (AMSTAR 2); Table S3 |
| Effect measures | 12 | Specify the effect measure(s) used. | Methods – Statistical analysis (eG, eOR) |
| Synthesis methods | 13a–13f | Eligibility for synthesis; data preparation; tabulation; synthesis model; heterogeneity; sensitivity analyses. | Methods – Statistical analysis |
| Reporting bias | 14 | Describe methods to assess risk of bias due to missing results. | Methods; Suppl. Methods 1.3 |
| Certainty assessment | 15 | Describe methods to assess certainty in the body of evidence. | Methods (Ioannidis classification / GRADE) |
| **RESULTS** |  |  |  |
| Study selection | 16a | Report numbers screened, assessed, and included (flow diagram). | Figure 1; Results |
| Study selection | 16b | Cite excluded studies that seemed to meet criteria, with reasons. | Table S2 |
| Study characteristics | 17 | Cite each included study and present its characteristics. | Tables 1–2 |
| Risk of bias in studies | 18 | Present risk-of-bias assessments for each included study. | Table S3 |
| Results of individual studies | 19 | Present summary statistics and effect estimates for each study. | Figures 2–3; Table S4 |
| Results of syntheses | 20a–20d | Summarise characteristics/risk of bias; present each synthesis with CIs and heterogeneity; sensitivity analyses. | Table 3; Figures 2–4; Table S4 |
| Reporting biases | 21 | Present assessments of risk of bias due to missing results. | Results – Heterogeneity; Table S4 (Egger, ESB) |
| Certainty of evidence | 22 | Present certainty (or confidence) assessments for each outcome. | Table 3; Table S4 |
| **DISCUSSION** |  |  |  |
| Discussion | 23a–23d | Interpretation; limitations of evidence; limitations of review process; implications. | Discussion |
| **OTHER INFORMATION** |  |  |  |
| Registration and protocol | 24a–24c | Registration name/number; where the protocol can be accessed; amendments. | Methods (PROSPERO CRD420261386392) |
| Support | 25 | Describe sources of financial/non-financial support and role of funders. | Role of the funding source; Funding |
| Competing interests | 26 | Declare any competing interests. | Declaration of interests |
| Availability of data/code | 27 | Report which data, code, and other materials are available and where. | Data sharing |

**3. Supplementary tables**

**Table S1. Full electronic search strategy, by database**

*Line-by-line search strategy for each of the six databases reported in the main text, run on 2 May 2026 (initial searches in PubMed and the Cochrane Database on 1 May 2026, then all databases re-run with the final strategy on 2 May 2026); date limits 1 January 2015 to 2 May 2026; languages English and Turkish*

| **Database (platform)** | **Search string (as run)** | **Date run** |
| --- | --- | --- |
| PubMed/MEDLINE | ("Aged"[Mesh] OR "Aged, 80 and over"[Mesh] OR "Frail Elderly"[Mesh] OR older adult*[tiab] OR elderly[tiab] OR geriatric*[tiab] OR aging[tiab] OR ageing[tiab] OR senior*[tiab] OR "old age"[tiab] OR "older people"[tiab] OR "older patient*"[tiab]) AND ("Telemedicine"[Mesh] OR "Mobile Applications"[Mesh] OR "Wearable Electronic Devices"[Mesh] OR "Text Messaging"[Mesh] OR "Cell Phone"[Mesh] OR "Smartphone"[Mesh] OR "Internet-Based Intervention"[Mesh] OR mHealth[tiab] OR "m-health"[tiab] OR "mobile health"[tiab] OR telehealth[tiab] OR telemedicine[tiab] OR eHealth[tiab] OR "e-health"[tiab] OR "digital health"[tiab] OR "digital intervention*"[tiab] OR smartphone*[tiab] OR "mobile app*"[tiab] OR "mobile phone*"[tiab] OR "cell phone*"[tiab] OR wearable*[tiab] OR "fitness tracker*"[tiab] OR "activity tracker*"[tiab] OR "text messag*"[tiab] OR SMS[tiab] OR "internet-based"[tiab] OR "web-based"[tiab] OR "online intervention*"[tiab]) AND ("Patient Education as Topic"[Mesh] OR "Self Care"[Mesh] OR "Self-Management"[Mesh] OR "Health Education"[Mesh] OR "Self Efficacy"[Mesh] OR "Health Literacy"[Mesh] OR "self-management"[tiab] OR "self management"[tiab] OR "self-care"[tiab] OR "self care"[tiab] OR "patient education"[tiab] OR "health education"[tiab] OR "self-efficacy"[tiab] OR "self efficacy"[tiab] OR "health literacy"[tiab] OR "behavior change"[tiab]) AND ("Meta-Analysis"[Publication Type] OR "Systematic Review"[Publication Type] OR "meta-analysis"[tiab] OR "systematic review"[tiab] OR "umbrella review"[tiab] OR "overview of reviews"[tiab]) | 2 May 2026 |
| CINAHL Complete (EBSCO) | (MH "Aged+" OR MH "Frail Elderly" OR TI ( "older adult*" OR elderly OR geriatric* OR aging OR ageing OR senior* OR "older people”) OR AB ( "older adult*" OR elderly OR geriatric* OR aging OR ageing OR senior* OR "older people" )) AND (MH "Telehealth" OR MH "Mobile Applications" OR MH "Wearable Sensor Technology" OR MH "Text Messaging" OR MH "Smartphone" OR MH "Telemedicine" OR TI ( mHealth OR "mobile health" OR telehealth OR telemedicine OR eHealth OR "digital health" OR smartphone* OR "mobile app*" OR wearable* OR "text messag*" OR SMS OR "web-based" OR "online intervention*" ) OR AB ( mHealth OR "mobile health" OR telehealth OR telemedicine OR eHealth OR "digital health" OR smartphone* OR "mobile app*" OR wearable* OR "text messag*" OR SMS OR "web-based" OR "online intervention*" )) AND (MH "Patient Education" OR MH "Self Care" OR MH "Self-Management" OR MH "Health Education" OR MH "Self-Efficacy" OR MH "Health Literacy" OR TI ( "self-management" OR "self-care" OR "patient education" OR "health education" OR "self-efficacy" OR "health literacy" OR "behavior change" ) OR AB (“self-management" OR "self-care" OR "patient education" OR "health education" OR "self-efficacy" OR "health literacy" OR "behavior change”)) AND (PT "Systematic Review" OR PT "Meta Analysis" OR TI ( "meta-analysis" OR "systematic review" OR "umbrella review" ) OR AB (“meta-analysis" OR "systematic review" OR "umbrella review" )) | 2 May 2026 |
| Cochrane Database of Systematic Reviews | #1: MeSH descriptor: [Aged] explode all trees  #2: ("older adult*" OR elderly OR geriatric* OR aging OR ageing OR senior*):ti,ab,kw  #3: #1 OR #2  #4: MeSH descriptor: [Telemedicine] explode all trees  #5: MeSH descriptor: [Mobile Applications] explode all trees  #6: MeSH descriptor: [Wearable Electronic Devices] explode all trees  #7: (mHealth OR "mobile health" OR telehealth OR telemedicine OR eHealth OR "digital health" OR smartphone* OR "mobile app*" OR wearable* OR "text messag*" OR SMS OR "web-based" OR "online intervention*"):ti,ab,kw  #8: #4 OR #5 OR #6 OR #7  #9: MeSH descriptor: [Self-Management] explode all trees  #10: MeSH descriptor: [Patient Education as Topic] explode all trees  #11: MeSH descriptor: [Self Efficacy] explode all trees  #12: ("self-management" OR "self-care" OR "patient education" OR "health education" OR "self-efficacy" OR "health literacy"):ti,ab,kw  #13: #9 OR #10 OR #11 OR #12  #14: #3 AND #8 AND #13 | 2 May 2026 |
| Epistemonikos | (elderly OR "older adult" OR aged OR geriatric) AND (mHealth OR telehealth OR "digital health" OR smartphone OR "mobile health" OR wearable OR "web-based") AND ("self-management" OR "self-care" OR "patient education" OR "health education") | 2 May 2026 |
| Web of Science Core Collection | TS=("older adult*" OR elderly OR geriatric* OR aging OR ageing OR senior* OR "older people")  AND TS=(mHealth OR "m-health" OR "mobile health" OR telehealth OR telemedicine OR eHealth OR "e-health" OR "digital health" OR "digital intervention*" OR smartphone* OR "mobile app*" OR "mobile phone*" OR wearable* OR "fitness tracker*" OR "text messag*" OR SMS OR "internet-based" OR "web-based" OR "online intervention*") AND TS=("self-management" OR "self-care" OR "patient education" OR "health education" OR "self-efficacy" OR "health literacy" OR "behavior change") AND TS=("meta-analysis" OR "systematic review" OR "umbrella review" OR "overview of reviews") | 2 May 2026 |
| Scopus | (TITLE-ABS-KEY ( "older adult*" OR elderly OR geriatric* OR aging OR ageing OR senior* OR "older people" ) AND (TITLE-ABS-KEY (mHealth OR "m-health" OR "mobile health" OR telehealth OR telemedicine OR eHealth OR "e-health" OR "digital health" OR "digital intervention*" OR smartphone* OR "mobile app*" OR "mobile phone*" OR wearable* OR "fitness tracker*" OR "text messag*" OR SMS OR "internet-based" OR "web-based" OR "online intervention*”)  AND (TITLE-ABS-KEY (“self-management" OR "self-care" OR "patient education" OR "health education" OR "self-efficacy" OR "health literacy" OR "behavior change”) )  AND (TITLE-ABS-KEY (“meta-analysis" OR "systematic review" OR "umbrella review" OR "overview of reviews" ) ) AND (PUBYEAR > 2014 AND PUBYEAR < 2027 ) AND (DOCTYPE ( re ) OR DOCTYPE ( ar ) ) | 2 May 2026 |

**Table S2. Studies excluded at full-text screening, with reasons (n = 19)**

*Nineteen full-text articles were excluded. Citations, DOIs, and documented reasons are compiled from the screening workbook.*

| **#** | **Excluded study** | **DOI** | **Reason** |
| --- | --- | --- | --- |
| 1 | Kampmeijer R, et al. The use of e-health and m-health tools in health promotion and primary prevention among older adults: a systematic literature review. BMC Health Serv Res. 2016. | 10.1186/s12913-016-1522-3 | Primary-prevention focus; no chronic-disease management outcome |
| 2 | Yi JY, et al. Self-management of chronic conditions using mHealth interventions in Korea: a systematic review. Healthc Inform Res. 2018. | 10.4258/hir.2018.24.3.187 | Population not restricted to adults aged ≥60 years (mean ≈50) |
| 3 | Tighe SA, et al. Toward a digital platform for the self-management of noncommunicable disease: systematic review of platform-like interventions. J Med Internet Res. 2020. | 10.2196/16774 | Population not restricted to adults aged ≥60 years (mean 57–65) |
| 4 | Nkhoma DE, et al. Digital interventions self-management education for type 1 and 2 diabetes: a systematic review and meta-analysis. Comput Methods Programs Biomed. 2021. | 10.1016/j.cmpb.2021.106370 | Population not restricted to adults aged ≥60 years (mean 51.6) |
| 5 | Shrivastava TP, et al. Mobile app interventions to improve medication adherence among type 2 diabetes mellitus patients: a systematic review. J Diabetes Sci Technol. 2023. | 10.1177/19322968211060060 | Population not restricted to adults aged ≥60 years (median 53.2) |
| 6 | Kruse CS, et al. Analyzing the effectiveness of mHealth to manage diabetes mellitus among adults over 50: a systematic literature review. J Multidiscip Healthc. 2023. | 10.2147/JMDH.S392693 | Population not restricted to adults aged ≥60 years (adults >50) |
| 7 | Heitkemper EM, et al. Do health information technology self-management interventions improve glycemic control in medically underserved adults with diabetes? J Am Med Inform Assoc. 2017. | 10.1093/jamia/ocx025 | Population not restricted to adults aged ≥60 years (mean 55) |
| 8 | Nkhoma DE, et al. Impact of DSMES app interventions on medication adherence in type 2 diabetes mellitus: systematic review and meta-analysis. BMJ Health Care Inform. 2021. | 10.1136/bmjhci-2020-100291 | Population not restricted to adults aged ≥60 years (mean 57.6) |
| 9 | de Barros KAAL, et al. Effect and usability of mobile health applications for medication adherence in patients with heart failure: a systematic review. Int J Med Inform. 2023. | 10.1016/j.ijmedinf.2023.105206 | Population not restricted to adults aged ≥60 years (mean 51–72) |
| 10 | Kamalumpundi V, et al. The efficacy of web- or mobile-based interventions to alleviate emotional symptoms in people with advanced cancer: a systematic review. Support Care Cancer. 2022. | 10.1007/s00520-021-06496-z | Population not older-adult specific (adults ≥18; emotional-symptom outcomes) |
| 11 | Murdock P, et al. Information and communication technologies for chronic disease self-management in adults aged 65 years and older: scoping review. Interact J Med Res. 2026. | 10.2196/60542 | Ineligible study design (scoping review) |
| 12 | Chalfont G, et al. Self-efficacy of older people using technology to self-manage COPD, hypertension, heart failure, or dementia at home: an overview of systematic reviews. Gerontologist. 2021. | 10.1093/geront/gnaa045 | Ineligible study design (overview of reviews / umbrella review) |
| 13 | Chu RYK, et al. Technology-enabled interventions promoting healthy ageing: an umbrella review. Discov Public Health. 2025. | 10.1186/s12982-025-01066-8 | Ineligible study design (umbrella review) |
| 14 | Babar ZUD, et al. Randomized controlled trials covering pharmaceutical care and medicines management: a systematic review of literature. Res Social Adm Pharm. 2018. | 10.1016/j.sapharm.2017.06.008 | Intervention outside scope (face-to-face pharmaceutical care; non-digital) |
| 15 | Thuy LT, et al. Updating interventions to enhance medication adherence among community-dwelling older adults with chronic diseases: a systematic review. Home Healthc Now. 2026. | 10.1097/NHH.0000000000001405 | Intervention outside scope (predominantly face-to-face; non-digital) |
| 16 | Cajamarca G, et al. Technologies for managing the health of older adults with multiple chronic conditions: a systematic literature review. Healthcare (Basel). 2023. | 10.3390/healthcare11212897 | Ineligible outcomes (no health-effect outcomes measured) |
| 17 | Jeong HS, et al. Efficacy of digital therapeutic applications for cognitive training among older adults with mild cognitive impairment or dementia. Psychiatry Res. 2025. | 10.1016/j.psychres.2025.116426 | Ineligible outcomes (cognitive-rehabilitation outcomes) |
| 18 | Albarqi MN. Exploring the effectiveness of technology-assisted interventions for promoting independence in elderly patients: a systematic review. Healthcare (Basel). 2024. | 10.3390/healthcare12212105 | Ineligible outcomes (‘independence’, not chronic-disease self-management) |
| 19 | Cho E, et al. The effectiveness of non-pharmacological interventions using information and communication technologies for behavioural and psychological symptoms of dementia. Int J Nurs Stud. 2023. | 10.1016/j.ijnurstu.2022.104392 | Ineligible setting (predominantly residential long-term care) |

**Table S3. AMSTAR 2 item-level appraisal of the included systematic reviews**

*Y, Yes; PY, Partial Yes; N, No; —, not applicable (No meta-analysis). Critical domains are items 2, 4, 7, 9, 11, 13, and 15 (marked *). The overall confidence rating follows the AMSTAR 2 algorithm and matches Table 1 of the main text.*

| **Review** | **1** | **2*** | **3** | **4*** | **5** | **6** | **7*** | **8** | **9*** | **10** | **11*** | **12** | **13*** | **14** | **15*** | **16** | **Overall** |
| --- | --- | --- | --- | --- | --- | --- | --- | --- | --- | --- | --- | --- | --- | --- | --- | --- | --- |
| Son 2020 | Y | N | Y | Y | Y | Y | N | Y | Y | N | Y | N | PY | Y | N | Y | Critically low |
| Allida 2020 | Y | Y | Y | Y | Y | Y | Y | Y | Y | Y | Y | PY | Y | Y | N | Y | Low |
| Wong 2022 | Y | Y | Y | PY | Y | N | N | Y | Y | N | Y | PY | Y | Y | Y | PY | Low |
| Yang 2022 | Y | N | N | PY | Y | Y | N | PY | PY | N | PY | N | N | Y | N | PY | Critically low |
| Lee 2023 | Y | Y | Y | PY | Y | N | N | PY | Y | N | Y | N | N | Y | PY | PY | Critically low |
| Zhang 2024 | Y | Y | Y | PY | PY | PY | PY | Y | Y | PY | Y | PY | Y | Y | Y | Y | High |
| Park 2025 | Y | N | Y | PY | Y | PY | PY | Y | Y | N | Y | PY | PY | PY | N | Y | Critically low |
| Wang 2025 | Y | Y | Y | PY | Y | Y | PY | Y | Y | PY | Y | PY | Y | Y | Y | Y | High |
| Tam 2022 | Y | N | Y | PY | Y | PY | N | Y | Y | N | Y | PY | Y | Y | N | PY | Critically low |
| Ni 2024 | Y | Y | Y | PY | Y | Y | PY | Y | Y | PY | Y | Y | Y | Y | Y | PY | High |
| Lippi 2023 | Y | Y | Y | PY | Y | Y | PY | Y | Y | N | — | — | Y | PY | — | Y | High |

**Table S4. Full evidence classification of all 44 meta-analytic outcomes**

*Complete re-estimated metrics from the metaumbrella analysis (REML). Equivalent effect size is Hedges’ g (eG) for continuous outcomes and equivalent odds ratio (eOR) for binary outcomes; Figure 2 of the main text displays all outcomes on a common equivalent Hedges’ g axis. No outcome met the criteria for Class I, II, or III. eG, equivalent Hedges’ g; eOR, equivalent odds ratio; k, contributing RCTs; N, total participants; PI, prediction interval; ESB, excess significance bias; ns, non-significant (p ≥ 0.05); IV, weak evidence (p < 0.05).*

| **Outcome** | **Review** | **k** | **N** | **Equivalent ES [95% CI]** | **p** | **I² %** | **95% PI** | **Egger p** | **ESB p** | **Class** |
| --- | --- | --- | --- | --- | --- | --- | --- | --- | --- | --- |
| **Heart failure (17 outcomes)** | | | | | | | | | | |
| All-cause readmission | Zhang 2024 | 4 | 1357 | eOR 0.64 [0.531, 0.76] | 7.37 × 10⁻⁷ | 0 | [0.475, 0.85] | 0.19 | 0.003 | **IV** |
| Medication adherence (score) | Zhang 2024 | 3 | 487 | eG 0.797 [0.427, 1.168] | 2.48 × 10⁻⁵ | 72.1 | [-0.653, 2.247] | 0.39 | 0.27 | **IV** |
| Medication adherence (rate) | Zhang 2024 | 11 | 2127 | eOR 1.28 [1.1, 1.49] | 0.001 | 80.8 | [0.782, 2.095] | 0.003 | 0.00099 | **IV** |
| HF-related hospitalisation | Ni 2024 | 12 | 1636 | eOR 0.72 [0.575, 0.903] | 0.005 | 21.2 | [0.433, 1.198] | 0.0044 | 0.19 | **IV** |
| Length of hospital stay | Son 2020 | 2 | 1076 | eG -0.166 [-0.287, -0.045] | 0.007 | 0 | [-0.949, 0.617] | — | 0.46 | **IV** |
| Mortality | Zhang 2024 | 4 | 1337 | eOR 0.57 [0.328, 1.008] | 0.053 | 21.2 | [0.149, 2.221] | 0.045 | 0.4 | ns |
| Hospitalisation | Allida 2020 | 3 | 894 | eOR 0.74 [0.52, 1.057] | 0.098 | 0 | [0.34, 1.616] | 0.14 | 0.77 | ns |
| Quality of life | Son 2020 | 2 | 1127 | eG -0.080 [-0.2, 0.04] | 0.192 | 0 | [-0.86, 0.7] | — | 0.73 | ns |
| All-cause hospitalisation | Ni 2024 | 6 | 1195 | eOR 0.74 [0.455, 1.215] | 0.237 | 81.5 | [0.161, 3.427] | 0.1 | 0.014 | ns |
| Self-efficacy | Allida 2020 | 1 | 29 | eG 0.363 [-0.385, 1.11] | 0.321 | — | only 1 study | — | — | ns |
| Readmission | Son 2020 | 2 | 818 | eG -0.212 [-0.637, 0.213] | 0.329 | 76.8 | [-4.64, 4.216] | — | 0.16 | ns |
| Emergency department visits | Son 2020 | 2 | 458 | eG -0.090 [-0.282, 0.101] | 0.356 | 0 | [-1.331, 1.151] | — | 0.68 | ns |
| Cardiovascular mortality | Ni 2024 | 3 | 878 | eOR 0.86 [0.585, 1.273] | 0.458 | 0 | [0.368, 2.025] | 0.7 | 0.67 | ns |
| Disease knowledge | Allida 2020 | 3 | 411 | eG 0.074 [-0.136, 0.283] | 0.491 | 0 | [-0.448, 0.595] | 0.61 | 0.68 | ns |
| All-cause mortality | Ni 2024 | 7 | 1189 | eOR 0.91 [0.674, 1.224] | 0.528 | 0 | [0.626, 1.318] | 0.57 | 0.7 | ns |
| Health-related quality of life | Allida 2020 | 4 | 942 | eG 0.062 [-0.201, 0.325] | 0.644 | 59.4 | [-0.729, 0.853] | 0.53 | 0.26 | ns |
| Self-care behaviour | Allida 2020 | 3 | 411 | eG -0.074 [-0.69, 0.541] | 0.813 | 86.5 | [-2.584, 2.436] | 0.63 | 0.0027 | ns |
| **Type 2 diabetes mellitus (5 outcomes)** | | | | | | | | | | |
| Postprandial glucose | Lee 2023 | 3 | 273 | eG -0.857 [-1.235, -0.478] | 9.36 × 10⁻⁶ | 57.4 | [-2.229, 0.516] | 0.7 | 0.31 | **IV** |
| Triglycerides | Lee 2023 | 6 | 1793 | eG -0.120 [-0.213, -0.028] | 0.011 | 0 | [-0.242, 0.001] | 0.84 | 0.51 | **IV** |
| Fasting blood glucose | Lee 2023 | 5 | 1623 | eG -0.222 [-0.465, 0.02] | 0.072 | 57.7 | [-0.88, 0.436] | 0.15 | 0.48 | ns |
| HbA1c | Yang 2022 | 9 | 4264 | eG -0.298 [-0.638, 0.041] | 0.085 | 92.2 | [-1.481, 0.885] | 0.064 | 0.001 | ns |
| HbA1c | Lee 2023 | 8 | 3047 | eG -0.824 [-1.966, 0.317] | 0.157 | 99.3 | [-4.935, 3.287] | 0.38 | 0.9 | ns |
| **COPD (8 outcomes)** | | | | | | | | | | |
| Quality of life — EQ-5D | Wang 2025 | 4 | 447 | eG 0.250 [0.059, 0.441] | 0.01 | 0 | [-0.06, 0.56] | 0.059 | 0.5 | **IV** |
| Dyspnoea | Wang 2025 | 13 | 1603 | eG -0.207 [-0.374, -0.041] | 0.015 | 58.7 | [-0.747, 0.332] | 0.33 | 0.3 | **IV** |
| Quality of life — CCQ | Wang 2025 | 2 | 394 | eG -0.210 [-0.407, -0.013] | 0.037 | 0 | [-1.49, 1.07] | — | 0.83 | **IV** |
| Quality of life — CAT | Wang 2025 | 7 | 1134 | eG -0.201 [-0.402, 0] | 0.051 | 62.7 | [-0.769, 0.367] | 0.18 | 0.00061 | ns |
| Quality of life — SF-36 MCS | Wang 2025 | 4 | 510 | eG 0.108 [-0.074, 0.29] | 0.243 | 0 | [-0.187, 0.404] | 0.41 | 0.73 | ns |
| Quality of life — CRQ | Wang 2025 | 3 | 265 | eG 0.215 [-0.159, 0.589] | 0.259 | 54.8 | [-1.122, 1.552] | 0.92 | 0.4 | ns |
| Quality of life — SGRQ | Wang 2025 | 9 | 1083 | eG -2.553 [-7.618, 2.513] | 0.323 | 93.3 | [-21.314, 16.209] | 0.033 | 1 | ns |
| Quality of life — SF-36 PCS | Wang 2025 | 4 | 510 | eG 0.058 [-0.138, 0.254] | 0.564 | 15.6 | [-0.322, 0.438] | 0.59 | 0.7 | ns |
| **Hypertension (4 outcomes)** | | | | | | | | | | |
| Systolic blood pressure | Tam 2022 | 6 | 1103 | eG -0.415 [-0.62, -0.21] | 7.32 × 10⁻⁵ | 65.6 | [-0.992, 0.162] | 0.28 | 0.68 | **IV** |
| Medication adherence | Tam 2022 | 3 | 693 | eG -0.640 [-1.094, -0.186] | 0.006 | 88.9 | [-2.529, 1.249] | 0.066 | 0.035 | **IV** |
| Uncontrolled blood pressure | Tam 2022 | 2 | 542 | eOR 0.33 [0.076, 1.386] | 0.129 | 91.8 | [0, 2479116.487] | — | 0.0074 | ns |
| Diastolic blood pressure | Tam 2022 | 6 | 1103 | eG -0.169 [-0.461, 0.123] | 0.257 | 85.5 | [-1.088, 0.751] | 0.36 | 0.034 | ns |
| **Multimorbidity (10 outcomes)** | | | | | | | | | | |
| Self-efficacy | Wong 2022 | 4 | 1415 | eG 0.188 [0.081, 0.296] | 5.98 × 10⁻⁴ | 0 | [0.014, 0.363] | 0.038 | 0.29 | **IV** |
| Quality of life | Wong 2022 | 5 | 2308 | eG 0.117 [0.032, 0.201] | 0.007 | 25.3 | [-0.003, 0.237] | 0.81 | 0.53 | **IV** |
| Physical activity (MVPA) | Park 2025 | 3 | 2929 | eG 0.075 [0.002, 0.148] | 0.044 | 0 | [-0.086, 0.236] | 0.36 | 0.81 | **IV** |
| Depression | Park 2025 | 3 | 514 | eG -0.688 [-1.457, 0.082] | 0.08 | 94.8 | [-3.974, 2.599] | 0.089 | 0.29 | ns |
| Hospital admissions | Wong 2022 | 7 | 1417 | eOR 0.69 [0.448, 1.071] | 0.099 | 52 | [0.222, 2.164] | 0.54 | 0.27 | ns |
| Hospital days | Park 2025 | 2 | 406 | eG -1.559 [-3.556, 0.438] | 0.126 | 98.4 | [-23.868, 20.75] | — | 0.31 | ns |
| Depression | Wong 2022 | 3 | 759 | eG -0.394 [-1.04, 0.252] | 0.232 | 89.3 | [-3.145, 2.357] | 0.54 | 0.67 | ns |
| General quality of life | Park 2025 | 2 | 548 | eG 0.212 [-0.211, 0.634] | 0.326 | 84 | [-4.274, 4.697] | — | 0.43 | ns |
| LDL cholesterol | Park 2025 | 2 | 2501 | eG -0.035 [-0.114, 0.043] | 0.38 | 0 | [-0.544, 0.473] | — | 0.67 | ns |
| Systolic blood pressure | Park 2025 | 2 | 2499 | eG 0.117 [-0.259, 0.492] | 0.543 | 71.7 | [-3.762, 3.995] | — | 0.79 | ns |

*The 95% prediction interval included the null for all 44 outcomes. Between-study heterogeneity (I²) ranged 0–99.3%. Egger’s test was not computed for outcomes with fewer than the required number of studies (shown as —).*

**Table S5. Primary-study overlap underlying the corrected covered area (CCA)**

*Number of primary RCTs contributed by each review (diagonal) and shared between review pairs (off-diagonal). Across the 10 quantitatively contributing reviews there were 102 unique primary RCTs from 109 citation occurrences; six RCTs appeared in ≥2 reviews and one appeared in three. These counts underlie Figure 4.*

**(a) RCTs contributed by each review**

| **Review [cluster]** | **Primary RCTs (k)** |
| --- | --- |
| Wang 2025 [COPD] | 26 |
| Ni 2024 [HF] | 15 |
| Zhang 2024 [HF] | 13 |
| Wong 2022 [Multi] | 13 |
| Lee 2023 [DM] | 9 |
| Yang 2022 [DM] | 9 |
| Park 2025 [Multi] | 9 |
| Tam 2022 [HTN] | 6 |
| Allida 2020 [HF] | 5 |
| Son 2020 [HF] | 4 |

**(b) Shared RCTs between review pairs**

| **Review A** | **Review B** | **Shared RCTs** |
| --- | --- | --- |
| Wang 2025 | Wong 2022 | 1 |
| Lee 2023 | Yang 2022 | 1 |
| Lee 2023 | Park 2025 | 1 |
| Yang 2022 | Park 2025 | 1 |
| Allida 2020 | Ni 2024 | 1 |
| Ni 2024 | Son 2020 | 1 |
| Ni 2024 | Zhang 2024 | 1 |
| Park 2025 | Wong 2022 | 1 |

**Table S6. Corrected covered area (CCA), overall and by disease cluster**

| **Evidence base** | **CCA** | **Interpretation** |
| --- | --- | --- |
| Overall (10 reviews) | 0.76% | Slight overlap (<5%) |
| Heart failure | 2.94% | Slight overlap (<5%) |
| Multimorbidity | 4.76% | Slight overlap (<5%) |
| Type 2 diabetes mellitus | 5.88% | Marginally above 5% threshold |
| COPD; Hypertension | – | Single review each; overlap not assessable |
